# The Effect of Space Travel on Bone Metabolism: Considerations on Today’s Major Challenges and Advances in Pharmacology

**DOI:** 10.3390/ijms22094585

**Published:** 2021-04-27

**Authors:** Shirley Genah, Monica Monici, Lucia Morbidelli

**Affiliations:** 1 Department of Life Sciences, University of Siena, I-53100 Siena, Italy; shirley.genah@student.unisi.it; 2 ASA Campus Joint Laboratory, ASA Research Division, Department of Experimental and Clinical Biomedical Sciences “Mario Serio”, University of Florence, I-50139 Florence, Italy; monica.monici@unifi.it

**Keywords:** space pharmacology, bones, osteopenia, microgravity, space medicine

## Abstract

Microgravity-induced bone loss is currently a significant and unresolved health risk for space travelers, as it raises the likelihood for irreversible changes that weaken skeletal integrity and the incremental onset of fracture injuries and renal stone formation. Another issue related to bone tissue homeostasis in microgravity is its capacity to regenerate following fractures due to weakening of the tissue and accidental events during the accomplishment of particularly dangerous tasks. Today, several pharmacological and non-pharmacological countermeasures to this problem have been proposed, including physical exercise, diet supplements and administration of antiresorptive or anabolic drugs. However, each class of pharmacological agents presents several limitations as their prolonged and repeated employment is not exempt from the onset of serious side effects, which limit their use within a well-defined range of time. In this review, we will focus on the various countermeasures currently in place or proposed to address bone loss in conditions of microgravity, analyzing in detail the advantages and disadvantages of each option from a pharmacological point of view. Finally, we take stock of the situation in the currently available literature concerning bone loss and fracture healing processes. We try to understand which are the critical points and challenges that need to be addressed to reach innovative and targeted therapies to be used both in space missions and on Earth.

## 1. Introduction

Today, it is well established that the space “exposome”—the set of environmental factors related to space to which an astronaut is constantly exposed during a mission—can significantly influence human physiology [1]. Among the factors that place humans most at risk in space are microgravity and ionizing cosmic radiations (galactic cosmic radiation or GCR), deriving from the sun and other celestial bodies or events [2]. The human body results from a very long evolution shaped by gravitational forces; bones are needed to support body weight and muscles to carry out movements that counteract gravity [3,4]. The cardiovascular system has also adapted; our body is mainly composed of fluids, and a complex and delicate balance of hydrostatic forces is required to flow blood against gravity and maintain a constant supply of oxygen to the brain [4,5]. The transition to a condition where the gravitational force lacks causes a long series of physiological changes and related disorders.

Moreover, gravity plays a pivotal role in cell biology, acting on cytoskeletal organization and cell structure. Cells can convert mechanical inputs into biochemical signals, initiating downstream signaling cascades in a process known as mechanotransduction. Therefore, any changes in mechanical loading, for example, those associated with microgravity, can consequently influence cell functionality and tissue homeostasis [6], leading to altered physiological conditions.

Physiological changes in microgravity can either be immediate or manifest in the long-term with some permanent effects that never resolve completely. Within the first 72 h upon exposure to weightlessness, certain physiological systems exhibit altered function. These include baroreceptor reflex, neurovestibular system, and gastrointestinal tract, leading to problems like nausea and vomiting, space motion sickness (SMS), sleep disturbances and headaches [7]. The majority of these systems will adapt to microgravity by resetting to a new equilibrium state within a relatively short time. Upon return to a gravitational field from space, the same rapid effects may be seen in reverse [8].

Many of these immediate indispositions can interfere with crewmember critical operational procedures associated with entering orbit or returning to Earth. The lack of readiness in managing certain situations due to adaptation disturbances can represent a risk for mission success, and it is necessary to find effective and rapid countermeasures, both pharmacological and otherwise.

Other physiological systems manifest weightlessness effects on a longer term (weeks to months). For short-duration missions, these changes may be minor or even undetectable. On longer flights, the effects can become more pronounced. The recovery of astronauts from these symptoms depends on the time of exposure to stress factors related to the space environment; the longer they stay, the longer the recovering time [8,9]. Among long-term effects on human physiology, we find muscle atrophy, bone demineralization and, therefore, alteration in calcium balance, immune function dysregulation, endocrine disorders, such as insulin resistance [10,11], and cardiovascular deconditioning that leads to orthostatic intolerance [12]. Currently, these physiological changes present major obstacles to long-term space missions. Therefore, it is fundamental to develop countermeasures aimed at preventing or slowing down the progression of these complications.

This review discusses alteration of human physiological parameters related to bone metabolism due to long-term weightlessness exposure and examines the effectiveness of both pharmacological and non-pharmacological countermeasures.

## 2. Bone Loss and Osteoporosis

Reduction in bone density related to microgravity is due to an imbalance of bone remodeling induced by changes in bone cells. Studies of ground-based simulated microgravity showed that morphology and functions of osteoblasts, osteoclasts, osteocytes and mesenchymal stem cells were all changed, suggesting that bone cells respond and adapt to the altered gravity condition by changing their morphology and functions.

Much research demonstrated that modeled microgravity increased osteoclast activity, receptor activator of nuclear factor-kappa-Β ligand (RANKL)-mediated osteoclastogenesis and bone resorption capacity [13,14,15]. Nabavi and colleagues showed that unloading causes many negative effects in osteoblasts that are likely due to signaling cascades from the cell surface to the nucleus [16]. According to their results, osteoblasts show an altered microtubule organization, reduction in the size and number of focal adhesions (FA) and FA proteins (paxillin, vinculin and zyxin). Unloading has been shown to disrupt F-actin stress fiber formation, and a significant expansion in cell area was observed in osteoblasts exposed to microgravity, related to the altered F-actin tensional forces and decreased mechanical stiffness. Additionally, a significant change in nuclear morphology also has been observed [16]. Therefore, the absence of gravitational force affects the normal distribution of major cytoskeletal networks and the overall geometry of osteoblasts, influencing parallel cell function.

Osteocytes are remarkably versatile cells and are involved in all aspects of skeletal biology, including the response to loading, regulation of bone turnover and the control of mineral metabolism [17,18]. These cells are housed individually in cavities (lacunae) located inside the mineralized bone and communicate with each other, and with bone surface cells, by gap junctions connecting dendritic-like cellular extensions housed in connecting canaliculi [19]. Osteocytes regulate bone remodeling by sensing, responding and translating mechanical stimuli into biochemical signals that affect other bone cell functions (e.g., osteoblast, osteoclast) [17,18]. In the presence of loading stimuli, osteocytes produce a typical signaling glycoprotein, sclerostin, which provides for negative regulation of bone formation by inhibiting the Wnt-β-catenin pathway in osteoblasts [20,21]. Usually, Wnt binding to its frizzled receptor complexed with low-density lipoproteins 5 or 6 (Lrp5/Lrp6) initiates a signaling cascade resulting in B-catenin translocating to the nucleus, where it activates transcription factors regulating target genes involved in osteoblast proliferation and differentiation [19,20,21]. The Wnt-signaling pathway is not only involved in osteoblastic cell differentiation and bone formation but also inhibits bone resorption by blocking the receptor activator of nuclear factor-κB-ligand (RANKL)/RANK interaction [1,22]. Many studies in the literature have reported that when reduced mechanical loading and microgravity occur, the production of sclerostin by osteocytes increases notably, blocking Wnt binding and thus resulting ultimately in reduced osteoblast and increased osteoclast activity [23,24,25].

Alongside this finding, recent data have demonstrated that osteocytes can remodel their perilacunar and canalicular matrix and participate in the release of skeletal calcium stores, a process called “osteocytic osteolysis”, which occurs in response to diverse stimuli [7]. Blaber et al. found that bone loss induced by microgravity in mice was not only due to osteoclastic hyperactivation but also caused by osteocytic osteolysis and osteoblastic cell cycle inhibition [26]. Taken all this evidence together, it can be hypothesized that osteocytes function in two ways to respond to microgravity. On one hand, they sense the gravity changes and begin osteocytic osteolysis themselves. On the other hand, they send signals to other effector cells (e.g., osteoclast, osteoblast) to regulate their functions.

Blaber and colleagues also proposed the hypothesis that at the basis of bone formation decrease, there is also an alteration in the differentiation of bone marrow mesenchymal and hematopoietic stem cell lineages [27,28]. The group conducted in vivo studies using 16-week-old female mice flown for 15-days on the International Space Station (ISS). It analyzed femoral bone marrows, which displayed large cavities, clearly indicating unloading-associated bone resorption, and demonstrated a general downregulation pattern of gene expression required to differentiate bone marrow mesenchymal stem cells (MSC) and hematopoietic stem cells (HSC) progenitor lineages. This phenomenon is revealed in multiple cell types, including osteoclasts and osteoblasts required for bone remodeling and mineral homeostasis, erythrocytes required for the transport of oxygen and iron throughout the body, and megakaryocytes required for the formation of platelets [27,28].

Microgravity-induced bone loss has been suggested to be more severe than osteopenia on Earth, and prolonged exposure to unloading conditions can raise the risk of osteoporosis and bone fractures [29]. Bone loss onset is usually very fast and can be detected within the first months in microgravity, whereas bone density recovery and re-mineralization are slower at 1 g on Earth and, in some cases, are not even restored to previous levels [30]. Bone demineralization during space flights also increases urinary calcium excretion and raises the risk of kidney stone formation. In physiological conditions, blood calcium and phosphate kinetics are regulated by the parathyroid gland through the secretion of parathyroid hormone (PTH), whose effects concern bone turnover, kidneys homeostasis and intestine Ca^2+^ absorption by stimulating the production of active vitamin D [31]. As a consequence of bone demineralization, due to unloading, Ca^2+^ released from bones reaches high blood levels causing suppression of PTH secretion. This inhibition leads to a reduction in vitamin D production at the intestine level, a decrease in kidney Ca^2+^ reabsorption and hypercalciuria, raising the risk of kidney stone formation [32].

Despite interindividual heterogeneity in bone mass, not all sites of the skeleton are similarly affected by unloading. Weight-bearing bones at 1xg are majorly affected by the space environment compared to non-weight-bearing bones. Indeed, the tibial cancellous bone showed more striking and earlier bone loss than the tibial cortex, probably because of more local remodeling [30]. According to National Aeronautics and Space Administration (NASA), the proximal femoral bone loses 1% to 1.5% of its mass per month or roughly 6% to 10% over a 6-month stay in space. Recovery after returning to Earth takes at least 3 or 4 years [33].

Microgravity-induced osteopenia is a significant and unresolved health risk for space travelers. For this reason, many countermeasures are employed today to reduce bone loss during long-duration space flights and include diet supplementations, physical activity and pharmacological interventions.

## 3. Countermeasures

### 3.1. Diet Supplements

Although the most important cause for bone loss in space is unloading of the body weight, when considering the long duration of spaceflight, there are several other factors able to influence calcium metabolism, including alterations in diet intake and low sunlight exposure [34]. Calcium and vitamin D have long been recognized as essential nutrients for bone health and maintenance.

The principal function of vitamin D in calcium homeostasis is to increase calcium absorption from the bowel. A deficiency of this element, due to poor exposure of astronauts to sunlight and ultraviolet radiations, may lead to insufficient Ca^2+^ intake, diminished intestinal absorption, decreased serum Ca^2+^ levels, which cause poor mineralization of bones [34]. Ca^2+^ intake is generally associated with the ingestion of specific foods, such as dairy products, nuts and seeds, vegetables like kale, broccoli and watercress and salmon. Unfortunately, despite many attempts to implement astronauts’ regimes, diet components are not sufficiently absorbed. Furthermore, the presence of intolerances to dairy products may play a negative role in assuming adequate calcium levels. Lactose intolerance has been associated not only with poor Ca^2+^ intake but also with low bone mass and increased risk of fracture [35]. In these cases, diet supplements are needed to reach optimal nutritional needs of calcium. Astronauts necessitate sufficient oral intake of vitamin D and Ca^2+^ during spaceflights, and the amount necessary to minimize a negative balance is approximately 1000 mg/d for calcium and 800–1000 IU/d for vitamin D, which is currently recommended for space flights up to one year [34,36].

### 3.2. Physical Exercise

Until 2004 early countermeasures on the ISS included an interim resistance exercise device (iRED), treadmills and exercise bicycle. In 2004, NASA installed the advanced resistance exercise device (aRED) with higher loads to increase forces on bone, particularly the spine and proximal femur [37]. Smith et al. showed in 2012 that resistance exercise coupled with adequate dietary supplements could maintain bone mass in most regions during short space missions. In this study, involving 13 crew members on ISS missions from 2006 to 2009, eight members had access to iRED, and five had access to the advanced resistive exercise device aRED. All crew members were provided with a specific program prescribing 2.5 h of exercise per day (6 days/week), including aerobic training and resistance exercise. Diet was designed to provide adequate intake of seven nutrients of interest (proteins, water, energy, sodium, calcium, iron, and potassium). The authors concluded that improved nutrition and resistance exercise during spaceflight could attenuate the expected bone resorption observed on prolonged missions [1,38].

### 3.3. Pharmacological Countermeasures

Physical activity and nutrient integration are usually coupled and associated with pharmacological therapies to prevent bone loss. Today, treatments for space osteoporosis include antiresorptive drugs aiming at actively reducing osteoclast numbers by inhibiting osteoclastogenesis or inducing apoptosis, thereby reducing resorption levels. The list of pharmacological countermeasures and related dosages is summarized in Table 1.

#### 3.3.1. Antiresorptive Agents

Among antiresorptive drugs, bisphosphonates are primary agents in the current pharmacological arsenal against osteoclast-mediated bone loss. Structurally, bisphosphonates are chemically stable derivatives of inorganic pyrophosphate. Second- and third-generation bisphosphonates (alendronate 70 mg, risedronate 35–75 mg, ibandronate 150 mg, pamidronate 60–90 mg, zoledronic acid 4 mg) have nitrogen-containing R2 side chains [39]. Bisphosphonates are preferentially incorporated into sites of active bone remodeling, and the primary pharmacological action is the suppression of bone resorption by inducing selective osteoclast apoptosis. Specifically, bisphosphonates attach to hydroxyapatite binding sites on bone surfaces undergoing active resorption [39,40]. When osteoclasts begin to resorb bone that is impregnated with a bisphosphonate, the released drug during resorption impairs the ability of the osteoclasts to form the ruffled border, to adhere to the bony surface, and to produce the protons necessary for continued bone resorption [39,40].

LeBlanc and colleagues studied the administration of alendronate as a supplement to exercise to protect bone during long-duration spaceflight. According to their results, the combination of the aRED exercises (2.5 h, 6 days/week) and oral administration of alendronate (Fosamax^®^ 70 mg/week) attenuated the expected decline in essentially all indices of altered bone physiology during spaceflight [41]. Today, the combination of aRED exercises, vitamin D and calcium supplementation together with bisphosphonates administration seems to be the best countermeasure to employ to prevent astronaut bone loss during space flights.

An interesting molecular target that could slow down bone resorption is RANKL (receptor activator of NF-kB ligand), a protein expressed by osteoblasts, bone marrow, stromal cells and chondrocytes. The interaction of RANKL with its receptor on osteoclasts RANK is involved in cell differentiation and activation [42]. The monoclonal antibody denosumab shows antiresorptive activity by selectively blocking the binding of RANKL to RANK on osteoclasts [43]. In the FREEDOM clinical trial, denosumab subcutaneous administration at the dose of 60 mg every six months was effective in reducing fracture risk in women with postmenopausal osteoporosis. Over 3 years, denosumab significantly reduced the risk of new vertebral fracture by 68%, nonvertebral fracture by 20% and hip fracture by 40% relative to placebo. Furthermore, denosumab also significantly improved bone mineral density at various skeletal sites, including the total hip, lumbar spine, femoral neck and trochanter bone [43,44]. For its proven efficacy and safety, denosumab is being considered by NASA as a potential countermeasure for bone loss due to microgravity and results from experiments regarding its employment in space are still pending.

Antiresorptive drugs are potent agents in preventing bone loss and reducing fracture risk. However, long-term use of those medications, such as what might be done in space travel, can produce rare but potentially serious adverse effects, such as osteonecrosis of the jaw and atypical femoral fractures [44,45]. When thinking about drug therapy, especially in conditions that could alter per se pharmacokinetic parameters, such as space environment [46], several variables must be considered. These include the safety profile, route of administration and frequency: in fact, a drug with an easy route of administration, such as oral or intranasal, could ensure better adherence to the drug regimen, while a reduced frequency in assumption could decrease the onset of undesirable effects due to a continuous and repeated exposure to the medication [47]. To meet this last requirement, a medication must demonstrate a prolonged pharmacological action and, to date, some drugs that fall into the bisphosphonate class possess this property; indeed, some of them, in addition to an oral route of administration, also has a weekly (alendronate or risedronate) or even monthly (pamidronate) dosage. In particular, zoledronic acid is a bisphosphonate generally indicated for the treatment and prevention of osteoporosis and Paget’s disease [48]. The frequency in its administration changes according to the type of pathology considered. For the prevention and treatment of osteoporosis, it corresponds to an infusion of 4–5 mg of zoledronic acid, respectively, every one or two years [49]. Although the route of administration is complicated and requires the intervention of specialized healthcare personnel, its unusual frequency in dosage may suggest its use as a single infusion pre-launch. The same reasoning could be applied to the monoclonal antibody denosumab, whose dosage schedule is a subcutaneous administration of 60 mg every 6 months [43,44].

#### 3.3.2. Anabolic Agents

Considering adverse effects produced by antiresorptive agents, the focus and medical interest are shifting to developing pharmacological countermeasures acting through different mechanisms of action. Among these, anabolic drugs exert their function by increasing bone formation rather than inhibiting resorption.

Currently available anabolic agents improve bone mass and reduce fractures through stimulation of the parathyroid hormone receptor-1 on osteoblasts and their precursors. In a healthy organism, PTH functions as an essential endocrine regulator of calcium and phosphate concentrations in the extracellular space, which is crucial for maintaining serum and urinary calcium levels within the physiological range [50]. The first anabolic agent approved by Food and Drug Administration (FDA) to treat osteoporosis in postmenopausal women and in men, who are at high risk for fracture, is recombinant teriparatide (rhPTH (Forteo^®^)) an analog of human PTH. Subcutaneous daily administration of 20 μg of teriparatide for 19 months to women with low bone mass and a history of prior fracture resulted in an almost 10% increase in vertebral bone mineral density (BMD). Usually, teriparatide is also administered in combination with vitamin D (400–1200 IU) and calcium (1000 mg) supplementation [51]. Many other studies confirm the efficacy of teriparatide in preventing bone fractures and increasing BMD in osteoporosis patients and safety, considering no severe adverse reactions have been reported after long-term administration [52].

Nowadays, the administration of teriparatide through daily subcutaneous injections seems to be the most employed method. However, in environments such as the one aboard the ISS, it perhaps may not be indicated due to the difficulty in administration, which could hamper the compliance of the therapeutic regime. For this reason, it would be appropriate to evaluate different routes of administration. Several studies in the literature have demonstrated that oral formulations at higher doses (2.5 and 5 mg) of teriparatide show an efficacy, pharmacokinetic and safety profile comparable to the subcutaneous formulation [53,54]. To enhance absorption and bioavailability of therapeutic peptides, a new delivery system has been developed by Altaani and colleagues [55] in a preliminary in vivo study, which relies on teriparatide encapsulation in oleic acid-based nanoemulsions to be administered orally.

As discussed in previous paragraphs, microgravity-induced bone loss and demineralization raises the risk for kidney stone formation, as elevated blood Ca^2+^ levels suppress PTH secretion manifesting with hypercalciuria [56,57]. Therefore, studies with teriparatide, as an additional countermeasure for circulating PTH fall in microgravity and hypercalciuria, would be interesting in future space missions.

#### 3.3.3. Combination and Sequential Therapies

The effect of teriparatide, as an anabolic agent aimed at increasing BMD, is confined to a well-defined therapeutic window. Among the factors that mitigate the bone-forming activity, it must be considered that PTH, in addition to the bone-forming effect exerted on osteoblasts and osteocytes, also displays an indirect resorptive action by stimulating osteoclasts through a well-known molecular mechanism, already described elsewhere [50,58]. Furthermore, PTH’s ability to stimulate new bone formation waned with time and repeated dosing [59]. Indeed, several studies have shown that long-term administration of teriparatide beyond 24 months leads to increased resorptive markers and to a reduction in pro-forming bone markers, shifting the balance in the opposite direction. For this reason, using hrPTH is limited to a maximum period of 2 years, beyond which the anabolic effect on bones declines in favor of a catabolic action [60,61].

Teriparatide therapeutic regime, described in the previous paragraph, may be compatible with the duration of the current missions since astronauts rarely stay on the ISS for more than a year. To date, only the cosmonaut Valery Polyakov currently holds the overall record for the longest space mission, having completed a stay of 438 days aboard the ISS [62]. The problem arises when evaluating the possibility of long-term space flights, such as future expeditions to Mars, where extensive use of teriparatide alone will not only be able to counteract microgravity resorptive effects but instead could cause a synergic catabolic activity alongside the one induced by microgravity, leading to a dangerous risk for human health. For long-term treatment of osteoporosis, researchers have focused their attention on developing combined and sequential drug therapies with both anabolic and antiresorptive mechanisms of action.

In 2014, a clinical trial (DATA study) on postmenopausal osteoporotic women showed how the combination of teriparatide, 20 μg daily, and denosumab, 60 mg every 6 months, for 24 months could increase BMD at the femoral neck, total hip and spine significantly more than either teriparatide or denosumab alone [63]. Soon after, an extension study followed, aimed at evaluating the effect of sequential therapies on osteoporosis: women originally assigned to 24-months of teriparatide received 24-months of denosumab; subjects originally randomized to 24-months of denosumab shifted to 24-months of teriparatide; and subjects, who originally received both drugs, received an additional 24-months of denosumab alone [64]. BMD continued to increase transitioning from teriparatide to denosumab, whereas switching from denosumab to teriparatide resulted in progressive or transient bone loss. According to the authors, 2 years of combined therapy followed by 2 years of denosumab alone is associated with the largest cumulative BMD increases at the hip and radius, an important clinical outcome since increases obtained were greater than any currently available therapy taken for a similar duration. The additive effect of these two drugs appears to be linked to the ability of denosumab to fully inhibit teriparatide-induced bone resorption but only partially inhibit anabolic bone formation [64]. This combined and sequential approach could be evaluated for its effectiveness in long-term space travels. Ground-based simulated studies are needed and should be carried out to validate the potential of this 4-year therapeutic regimen. However, observations in real space conditions could be difficult due to the excessively long time astronauts should spend on the ISS. To date, no information is available regarding the effects of space exposome on human physiology over such prolonged times. Furthermore, it is unclear what effect stopping this therapy may have once the astronaut returns to normal gravity conditions.

### 3.4. Melatonin

All pharmacological countermeasures described above are effective in reducing bone loss associated with unloading conditions. However, they show some undesirable effects, even serious ones, which may arise after long-term use. For this reason, efforts have been made to find more riskless drugs for the prevention of bone loss during space flight. In this regard, researchers have focused their attention on melatonin, a hormone produced by the pineal gland, synthesized almost exclusively in the dark. Melatonin is known for its wide variety of physiologic functions, including hypothalamic control of circadian rhythms, body temperature, bone homeostasis and displays effects on both cardiovascular and immune systems [65,66,67]. The implication of melatonin in the maintenance of skeletal apparatus physiology has been raised by several studies associating a decrease in the nocturnal production of melatonin, due to aging or to light exposure at night, with increased risk of osteoporosis [68,69]. The effects of melatonin on bone marker turnover were further supported in the MelaOst trial demonstrating a significant improvement in BMD in postmenopausal women with osteopenia following nightly melatonin administration at the dose of 3 mg/day, for one year [69,70].

**Figure 1:**
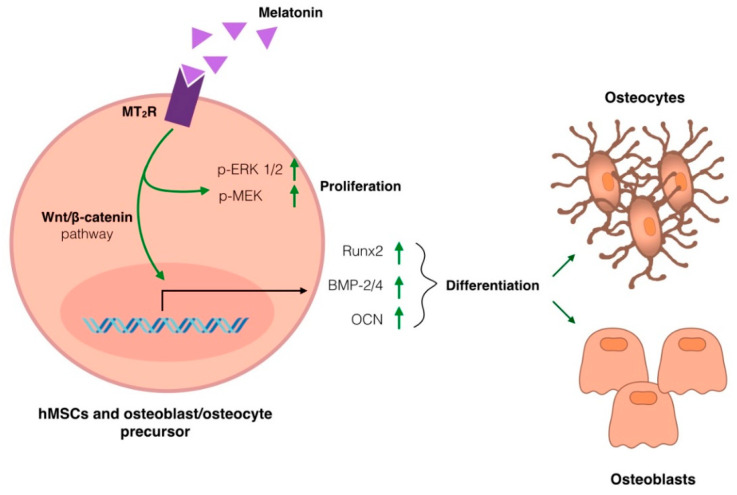
Molecular mechanism underlying melatonin’s anabolic effect on bone density. Melatonin binding to its G-protein coupled receptor (MT_2_R), located on the cell membrane of hMSCs and pre-osteoblasts, induces a signaling cascade that leads to the phosphorylation of MEK and ERK1/2 and, favoring (green arrows) cell proliferation and the upregulation of factors involved in cell differentiation via Wnt/β-catenin signaling pathway. The factors involved in controlling the bone formation and osteoblasts and osteocyte differentiation from precursor cell lines are bone morphogenetic proteins 2 and 4 (BMPs), runt-related transcription factor 2 (Runx2) and osteocalcin (OCN).

Today, molecular mechanisms responsible for melatonin anabolic effects on bone density have been extensively investigated and can be summarized in Figure 1. Briefly, melatonin can act on osteoblasts, favoring proliferation and differentiation, and simultaneously manifest inhibitory effects on osteoclast differentiation and bone-resorbing activity [65,69]. Melatonin binding to its G-protein coupled receptor (melatonin receptor 2 or MT2R), located on the cell membrane of hMSCs and pre-osteoblasts, induces a signaling cascade that leads to the activation, by phosphorylation, of MEK and ERK1/2, favoring cell proliferation and the upregulation of factors involved in cell differentiation via Wnt/β-catenin signaling pathway [65,66]. These factors, such as bone morphogenetic proteins 2 and 4 (BMPs), runt-related transcription factor 2 (Runx2) and osteocalcin (OCN), display anabolic action as they are positively involved in controlling the bone formation and osteoblasts and osteocyte differentiation from precursor cell lines [65,66,69].

**Figure 2:**
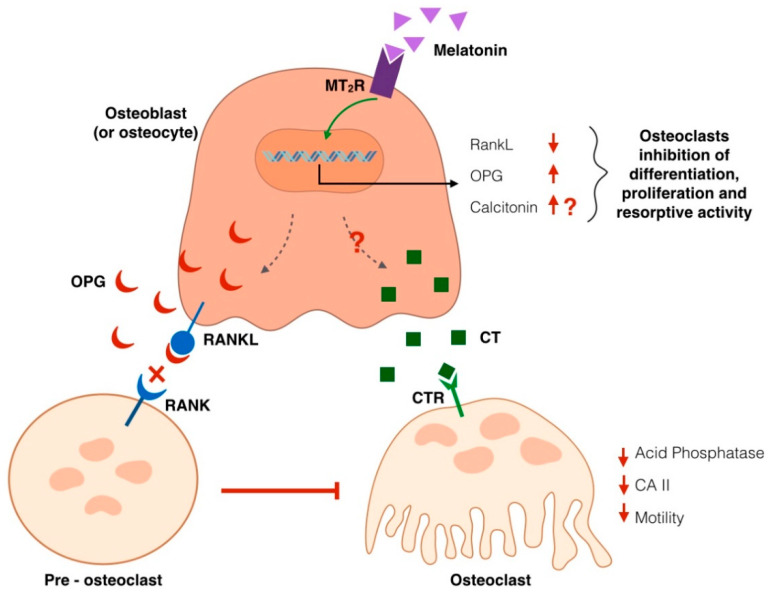
Molecular mechanism proposed for melatonin’s antiresorptive effect on bone density. Melatonin binding to its G-protein coupled receptor (MT2R), located on the cell membrane of osteoblasts/osteocytes and mesenchymal cells, induces a signaling cascade culminating in the modulation of genes that influence osteoclast differentiation and activity; specifically, a downregulation of RANKL and upregulation of osteoprotegerin (OPG), a decoy receptor produced by stromal cells as negative feedback to control osteoclastogenesis. Melatonin has also been proposed to produce an upregulation of calcitonin, whose binding to its receptor (calcitonin binding receptor or CTR) on osteoclasts induces a rapid cell contraction, causes inhibition of osteoclast motility. Calcitonin also inhibits (red arrows) other pathways associated with osteoclast activity, such as the release of acid phosphatase and the expression of carbonic anhydrase II (CA II), a cytosolic enzyme involved in the maintenance of an acidic environment, necessary for osteoclast resorption.

Recent literature also unraveled the mechanism underlying melatonin’s antiresorptive effects (Figure 2). In bone tissue, differentiation and activation of osteoclasts are generally influenced by interactions with osteoblastic lineage cells. Osteoclasts express the receptor activator for NFkB (RANK), which is a receptor for RANKL found on osteoblasts and bone marrow cells and is required for osteoclastogenesis and osteoclast activation [71,72]. RANKL signaling is usually inhibited by osteoprotegerin (OPG), a decoy receptor produced by stromal cells, as negative feedback to control osteoclastogenesis. Therefore, the ratio between RANKL:OPG expression is critical in this process [71,72]. Melatonin intracellular signaling leads to the modulation of genes that influence osteoclast differentiation and activity; specifically, a downregulation of RANKL and upregulation of OPG have been observed in cell culture studies after stimulation with melatonin, hence shifting the RANKL:OPG ratio towards an anti-osteoclastogenic activity [69]. This finding is also supported by clinical trials showing that in women treated with melatonin supplements, ratios of type-I collagen crosslinked N-telopeptide NTX (a bone resorption marker) to OCN trended downward compared to placebo. This is an important finding because as women transition through menopause, the NTX:OCN ratio increases such that osteoclast activity outpaces osteoblast activity leading to bone loss [73]. Recent studies on fishbone scales and chick calvariae models demonstrated that melatonin is also able to upregulate, in osteoblasts and their precursors, the transcription and production of another hormone, calcitonin, involved in bone remodeling [74,75]. However, more in-depth studies must be performed to verify this pattern also in human bone cells.

Calcitonin is a 32-amino acid linear polypeptide usually produced in humans primarily by the parafollicular cells of the thyroid gland. It acts as an osteoclast-inhibiting hormone, and today, salmon calcitonin is a currently FDA-approved medication as a second-line drug for postmenopausal osteoporosis and as a first-line treatment for hypercalcemia and Paget’s illness [76,77]. Focusing on calcitonin antiresorptive activity, the binding of this hormone to its receptor (calcitonin binding receptor or CTR) induces a rapid cell contraction and, therefore, causes inhibition of osteoclast motility which negatively affects cell capacity to resorb bone surfaces. However, this effect is temporary, and osteoclasts have been shown to gradually escape this inhibition after several hours [78,79,80]. Besides its action on cell motility, calcitonin also inhibits other pathways associated with osteoclast activity, such as the release of acid phosphatase and the expression of carbonic anhydrase II, a cytosolic enzyme involved in the maintenance of an acidic environment, necessary for osteoclast resorption [79,80,81]. Calcitonin also has been demonstrated to interfere with osteoclast differentiation from precursor cells and the fusion of mononucleated precursors to form multinucleated osteoclasts in bone marrow cultures [79,80].

Considering evidence indicating melatonin as a new therapeutic approach for the treatment of osteoporosis on Earth, through its dual anabolic and antiresorptive action, its use in space medicine could be advantageous for the treatment not only of osteopenia related to microgravity but also for the reestablishment of all those alterations linked to circadian rhythms. It is known. Indeed, changes in lighting and work schedules during spaceflight missions can impact circadian clocks and disrupt sleep, especially in the early stages of adaptation to living conditions on the ISS, hence compromising the mood, cognition and performance of orbiting astronauts [82,83]. To date, using melatonin in space has been mainly considered as a non-pharmacological remedy for the treatment of circadian misalignment and sleep deficiency. However, its clinical use for the treatment of microgravity-induced osteoporosis was first hypothesized by Ikegame and colleagues in 2019, thanks to their studies on interactions between osteoclasts and stromal cells in real microgravity conditions, using a new experimental bone model based on goldfish scales flown on the ISS [74]. Their interesting studies provide evidence that melatonin suppresses osteoclast bone-resorbing activity in bone tissues under microgravity conditions via the upregulation of calcitonin and the downregulation of RANKLin osteoblasts. Taking together all the aspects described in the previous paragraph, a more in-depth study of the dual, anabolic and antiresorptive effect of melatonin on bone metabolism is, therefore, highly encouraged in both simulated and real microgravity conditions.

## 4. Fractures and Bone Healing

In the previous paragraphs, the effect that the loss of bone mass can cause on the resistance and strength of the entire skeletal system has been brought to light several times. Indeed, one of the main consequences of severe bone mass loss and demineralization due to unloading conditions is the raised risk of both vertebral and nonvertebral fractures. Astronauts spending six months in spaceflight have on average a 10% loss in BMD, which is 10-fold greater than the BMD loss observed in postmenopausal women on Earth [1,24]. Beyond fractures due to progressive osteopenia induced by microgravity, it must also be considered that traumatic injuries, such as bone fractures and wounds, can naturally occur during routine operational procedures or extravehicular activities (EVA) [84,85]. Despite the evident medical need, however, the systemic response to fracture injury and the mechanism of human bone repair in unloading conditions is poorly investigated.

In normal conditions, tissue regeneration involves a coordinated interaction of cells, proteins, proteases, growth factors, small molecules and extracellular matrix (ECM) components to restore tissue morphology and functioning. Just like skin repair, also the bone healing process can be divided into multiple steps, and a communication network between stromal, endothelial, bone and immune cells is very important in determining the course of healing and recovery of tissue function [86,87,88]. Briefly, the first response to a bone fracture is constriction of the injured blood vessels and activation of platelets that form a fibrin clot (or hematoma) to cease blood flow and provide a scaffold for incoming cells by releasing signaling and growth factors, which, in turn, activate the migration of inflammatory cells and repair cells, such as fibroblasts, osteoblasts, stem cells and vascular endothelial cells [88]. The recruited fibroblasts begin to lay down the stroma that helps support vascular ingrowths while the responding macrophages remove tissue debris. Inflammatory cells also release growth factors and cytokines signals to recruit mesenchymal stem cells (MSCs), which proliferate and differentiate into osteoprogenitor cells and then into osteoblasts and osteoclasts to form and remodel newly formed bone tissue [86,87]. Much evidence in the literature highlights the pivotal role of blood vessels in the process of bone repair and osteogenesis, which indicates intimate molecular crosstalk between endothelial cells and osteoblasts [88,89]. Angiogenesis is required at different steps and, among the key roles played in the process, it furnishes oxygen and nutrient supply for the regenerating tissue, while endothelial cells (ECs) secrete osteogenic growth factors [90] to promote osteogenesis and osteoblast differentiation from their precursors. Newly formed blood vessels ensure steady transport of circulating osteoclast and osteoblast precursors to remodeling sites, regulating, therefore, osteoprogenitor cell invasion [90,91].

Many studies in the literature have reported that cell lines responsible for bone repair (MSC, fibroblasts, macrophages, osteoblasts, osteoclasts, and ECs), taken individually, are highly sensitive to microgravity and undergo morphological, functional, and biochemical changes under these conditions [16,27,92,93]. Besides osteoblast, osteoclast, and MSC alterations discussed previously, simulated microgravity studies have shown that ECs undergo important cytoskeletal remodeling and show impairment in proliferation and survival. However, there are still controversial data about ECs capacity to migrate and organize in 3D structures, and therefore, to sustain full angiogenic responses in unloading conditions [94], especially in the processes of wound or fracture healing.

Given the above, it could be deduced that if individual cell types manifest alterations in their morphology and function, the entire regeneration process may somehow be compromised by the lack of canonical gravitational forces. To date, very limited data in this regard is available. However, all studies performed on murine or rat models, both in real and simulated microgravity conditions, provide strong evidence and convey the same conclusion that the fracture healing is compromised in microgravity conditions and manifests in both histological and morphometric alterations and differences between animals healed in space or on Earth [95,96,97]. While these data demonstrate that microgravity has a deleterious effect on bone healing, the direct translation of these results to human bone healing is difficult due to the numerous differences between rodent and human bone microstructure and healing process. Furthermore, the molecular crosstalk among these cell lines in bone regeneration following fracture injuries is not fully known in conditions of real or simulated microgravity, and it is not yet clear how these physiological and key interactions can change. In vitro experiments using simulated µG devices or in vivo studies are, therefore, highly encouraged as elucidating molecular mechanisms at the basis of altered interaction between bone cells, endothelial and immune cells, in microgravity, could lead to the identification and development of specific countermeasures, both pharmacological or otherwise like bandages or specific dressings biomaterials, to improve and accelerate the regeneration process in space [98].

Besides studying the phenomenon from a molecular point of view, it would also be interesting to evaluate whether nonsurgical options currently in use on Earth to promote and facilitate bone regeneration in patients with healing deficiencies could be applied to space medicine for the treatment of mild fractures, which, in long-term spaceflights, could compromise astronaut performance. Among nonsurgical treatments, we find physical stimulation therapies, such as extracorporeal shockwave treatment (ESWT), low-intensity pulsed ultrasound (LIPUS) or low-level laser therapies (LLLT) [99,100,101,102]. The advantage of using nonsurgical countermeasures would lie in their non-invasive nature and easy application, which could allow the astronaut, in the event of minor fractures, rapid self-medication.

Advances in regenerative medicine are focused on the employment of mesenchymal stem cells (MSCs) to improve bone regeneration with interesting results due to their self-renewal and differentiation capacity and their ability to secrete bioactive molecules and regulate the behavior of other cells in different host tissues [103]. Instead, other research lines are evaluating pharmacological approaches based on the regulation of molecular mechanisms, such as antagonists of the WNT pathway [104,105], or using bone tissue morphogenic factors [106,107]. However, these lines of research have yet to find a consolidated use for the treatment of bone healing deficiencies on Earth, and their employment in space medicine will be subsequently validated.

## 5. Conclusions

Bone plays an important role as a structure that supports the body and represents a mineral reservoir for calcium and phosphate. The skeletal apparatus is in continuous reshaping, and its homeostasis is finely tuned by a balance in bone resorption and formation operated by osteoblasts, osteoclasts and osteocytes [3,4]. In a microgravity environment, because of reduced loading stimuli, this equilibrium is lost, and bone resorption prevails on bone formation, leading to bone mass loss at a rate of about ten times that of Earth osteoporosis [1,24]. Microgravity-induced osteopenia is, therefore, a significant and unresolved health risk for space travelers, which leads to a raised likelihood for irreversible changes that weaken skeletal integrity and to an increment in the onset of fracture injuries and renal stones formation [84,85].

Today several pharmacological and non-pharmacological countermeasures to this problem have been proposed, including physical exercise, diet supplements and administration of antiresorptive (Bisphosphonates or denosumab) or anabolic drugs (teriparatide). However, each class of pharmacological agents presents several limitations as prolonged and repeated employment of both antiresorptive and anabolic agents singularly is not exempt from the onset of serious side effects, which limit their use within a well-defined therapeutic window. Antiresorptive and anabolic drug therapeutic schedules, described in previous paragraphs, may be compatible with permanence in space comparable to that of the current missions since astronauts rarely stay on the ISS for more than a year. The problem arises when evaluating the possibility of long-term space flights (>2 years), such as future expeditions to Mars, where extensive and repeated use of antiresorptive agents may produce potentially serious adverse effects, such as osteonecrosis of the jaw and atypical femoral fractures [44,45], and anabolic drugs (teriparatide) could cause a synergic bone catabolic activity alongside to the one induced by microgravity, leading to a dangerous risk for human health [60,61].

For long-term treatment of osteoporosis on Earth, researchers have focused their attention on developing sequential drug therapies with both anabolic and antiresorptive mechanisms of action, which could prove to be very effective and useful in future exploration missions. In particular, recent clinical trials [63,64] demonstrated that two years of combined therapy (teriparatide + denosumab) followed by two years of denosumab alone is associated with the largest cumulative BMD increases at the hip and radius. However, the potential use of this pharmacological approach in space flights has yet to be validated through studies in real or simulated microgravity conditions. Furthermore, the 4-year therapeutic regimen applied in clinical studies on Earth to treat menopause osteoporosis is hardly applicable to observations in real space conditions due to the excessively long time astronauts should spend on the ISS. For this reason, the search for both safe and effective drugs for the long-term treatment of microgravity-related osteopenia, given longer space expeditions, remains an open challenge that requires major efforts to be resolved.

In the last decade, several studies have brought to light the role of melatonin in regulating and maintaining skeletal apparatus physiology. Today, the molecular mechanism at the basis of melatonin anabolic effects on bone density have been extensively investigated and can be summarized in a dual opposed action of both anabolic and antiresorptive activity: on one side, melatonin favors cell proliferation and osteoblasts and osteocyte differentiation from precursor cell lines; on the other hand, it hampers osteoclast differentiation [65,69]. Considering evidence indicating melatonin as a new therapeutic approach for the non-pharmacological treatment of osteoporosis on Earth, its use in space medicine, under the form of dietary supplement alongside vitamin D and calcium intake, could be advantageous for the treatment not only of osteopenia related to microgravity but also for the reestablishment of all those alterations linked to circadian rhythms due to changes in lighting and work schedules during spaceflight missions, that could compromise mood, cognition and performance of orbiting astronauts [82,83]. Preliminary data that emerged in the last years, and reported in this review, are definitely encouraging, but stronger clinical data in this regard are still lacking and are highly needed.

Another problem related to bone tissue homeostasis in microgravity is its capacity to regenerate following bone fractures due to both weakening of the tissue, caused by unloading related BMD loss, and accidental events, for instance, during the accomplishment of particularly complex operations. Following the above discussion, it has emerged that today there are still considerable gaps in this regard, and it is not known how human bone tissue responds during wound regeneration both at the molecular/cellular level, analyzing the crosstalk among these cell lines involved the process, and at the anatomical–morphological level. Gathering more detailed information on these processes in microgravity conditions could result advantageous for the identification and development of specific countermeasures, both pharmacological and otherwise. Therefore, we strongly believe that further studies must be performed in this direction, especially in view of long-term space operations, where reentry on Earth for bone trauma handling and care is highly unlikely.

## Figures and Tables

**Table 1:** List of pharmacological countermeasures, related dosages and administration routes proposed for the treatment of microgravity-induced osteopenia.

Activity	Class	Drug	Dosage	Administration
Anti-resorptive agents	Bisphosphonates	Alendronate	70 mg/week	Oral
		Risedronate	35–75 mg/week	Oral
		Ibandronate	150 mg/month	Oral
		Pamidronate	60–90 mg/month	Intravenous
		Zoledronic acid	4–5 mg/year	Intravenous
	Monoclonal antibodies	Denosumab	60 mg/6 months	Subcutaneous
Anabolic drugs		Teriparatide	20 μg/day	Subcutaneous
		2.5–5 mg/ day	Oral	
